# Efficacy and safety of everolimus treatment in a hemodialysis patient with metastatic atypical bronchial carcinoid: case report and literature review

**DOI:** 10.1186/s12885-018-4205-0

**Published:** 2018-03-20

**Authors:** M. P. Brizzi, A. La Salvia, M. Tampellini, C. Sonetto, M. Volante, G. V. Scagliotti

**Affiliations:** 1 Medical Oncology, Department of Oncology, University of Turin, Azienda Ospedaliera Universitaria San Luigi, Regione Gonzole 10, 10043, Orbassano, Turin Italy; 2 Department of Medical Oncology and Pathology, University of Turin, Azienda Ospedaliera Universitaria San Luigi, Regione Gonzole 10, 10043, Orbassano, Turin Italy

**Keywords:** Bronchial carcinoids, Everolimus, Hemodialysis, Pharmacokinetics, Safety

## Abstract

**Background:**

Everolimus was recently approved for the treatment of neuroendocrine tumors. However, its efficacy and tolerability in hemodialysis patients with end-stage renal disease is not established.

**Case presentation:**

We describe the case of a 47-year-old man with end-stage renal disease who received everolimus plus Lanreotide for 9 months for the management of metastatic atypical bronchial carcinoid.

**Conclusions:**

Everolimus is a treatment option for hemodialysis patients with metastatic atypical bronchial carcinoid. Based on our case report and review of literature, Everolimus does not require any dose reductions and is overall well tolerated in hemodialysis patients.

## Background

In two phase III trials everolimus has been shown to be active against neuroendocrine tumors (NETs) arising from the lung [1, 2]. Its tolerability in lung NET patients with concomitant severe renal insufficiency has never been reported. Here we describe the case of a hemodialysis patient with metastatic atypical bronchial carcinoid treated with everolimus.

## Case presentation

In February 2011, a 47-year-old white male patient underwent right lung lobectomy for resection of an atypical bronchial carcinoid (pT1pN2). His medical history was significant for end-stage renal disease (ESRD) secondary to focal segmental glomerulosclerosis requiring iterative hemodialysis. In September 2011 a right hepatectomy was performed due to liver metastases; metastatic disease progressed to liver and bone over the next 17 months. Monthly administration of intramuscular Lanreotide 120 mg was initiated and continued for 1 year. In March 2014 further disease progression was noted and oral everolimus was initiated at a dose of 10 mg/day, then reduced to 5 mg/day 1 month later due to persistent grade II mucositis. No grade III-IV toxicities occurred. A computed tomography (CT) scan taken 3 months later showed stabilization of disease. Everolimus therapy was discontinued in November 2014, after a 9-month course of treatment, because of hepatic and bone disease progression. Somatostatin analogue therapy was continued throughout. A CT scan taken 6 months later revealed progression of liver disease and development of peritoneal metastases, for which 5 cycles of chemotherapy with temozolomide were administered from April to August 2015. Liver metastases and peritoneal metastases progressed, and oral metronomic chemotherapy with capecitabine was initiated in November 2015. The patient died in January 2016 due to disease progression.

## Discussion and conclusions

Several therapeutic options are available for front-line treatment of metastatic bronchial carcinoids, including somatostatin analogues, chemotherapy, and peptide receptor radionuclide therapy [3].

However, comorbidities such as renal failure can limit or even preclude such treatments [4].

Unfortunately, there are no established guidelines for the administration of chemotherapeutic or target agents in hemodialysis patients [5]. The prevalence of renal insufficiency in cancer patients is growing [6], making observations in any tumor setting important.

To the best of our knowledge, this is the first report of a hemodialysis patient with metastatic atypical bronchial carcinoid treated with everolimus. In the one case of a hemodialysis patient with metastatic ileal well differentiated NET (G1) described so far, disease stabilization was achieved without any adverse effects [7].

Clinical trials investigating everolimus activity have excluded ESRD patients because the condition is thought to alter drug pharmacokinetics. Indeed, drug clearance may be different in hemodialysis patients, increasing the risk of overdosage and higher toxicities [8], or it may be more rapid and thus reduce drug efficacy. Recent reports have documented that everolimus pharmacokinetics is not altered by hemodialysis in patients with metastatic renal cell cancer [9–13], probably owing to the absence of diffusion of everolimus through commonly used dialysis membranes and in the dialysate fluid (Table 1). Another explanation for the unchanged tolerability profile of everolimus in patients with renal insufficiency is that the drug is predominantly metabolized by the liver and eliminated in the bile [14].

**Table 1:** Everolimus in the treatment of hemodialysis patients with metastatic renal cell carcinomas

Authors/reference	Study description	No. of patients included	No. of prior regimen	Everolimus dosage	Best responses	Duration of response	Safety
Thiery-Vuillemin A et al. [9]	Case reports	2	1; 1	5 mg	NA	NA	Grade II asthenia, diarrhea and mucositis; grade III hyperglycemia
Syrios J et al. [10]	Case reports	2	1; 1	10 mg	SD; CR	4 mo; 40 mo	Well tolerated
Adytia V et al. [11]	Single institution experience	6	NA	10 mg; 1 dosage reduction to 5 mg	SD	Median duration of therapy 1.9 mo (0.4–17)	5 patients: well tolerated, 1 patient: pneumonitis
Czarnecka AM et al. [12]	Single institution experience	1	2	10 mg	PD	4 mo	Well tolerated
Omae K et al. [13]	Single institution experience	4	1		SD	Median duration of therapy 6.7 mo	Grade II rash, diarrhea, pneumonitis, and mucositis

*Abbreviations: NA* Not available, *SD* Stable disease, *PD* Progression of disease, *mo* Months

Moreover, repeated surgery for multiple renal cell carcinoma in patients with von Hippel-Lindau syndrome may lead to ESRD in up to 25% of cases [15]. In these young patients, pancreatic NETs occurrence is frequent. Treatment with everolimus may be considered safe in this patient setting. Everolimus therapy is feasible in hemodialysis patients with metastatic bronchial carcinoid. Its observed efficacy and tolerability profile are similar to those reported in patients with normal renal function. To confirm the clinical features of everolimus in NET patients undergoing hemodialysis, data collection from single case or case series is highly envisioned. This will remove any concern about treating this rare patient subgroup with an active treatment allowing their inclusion in prospective trials.

## Abbreviations

CT: Computed tomography
ESRD: End-stage renal disease
G1: Grade 1
Mg: Milligrams
NET: Neuroendocrine tumor
NETs: Neuroendocrine tumors
pT1pN2: UICC pathological TNM staging system
